# Meta Analysis of DHA and EPA Supplementation on Cardiovascular Outcomes and Atrial Fibrillation Risk

**DOI:** 10.1002/prp2.70265

**Published:** 2026-05-17

**Authors:** Sepideh Karkon Shayan, Rosull Saadoon Abbood, Sarah F. Al‐Taie, Paria Ganji Nataj, Maksudova Malika Khamdamjonovna, Siamak Aminnezhad, Yasaman Ghodsi Boushehri, Jabbarov Jamoliddin Sindorovich, Ali Sinehsepehr, Farshad Zare, Seyed Abbas Pakmehr, Elham Abdollahi, Milad Vahedinezhad

**Affiliations:** ^1^ School of Medicine Gonabad University of Medical Sciences Gonabad Iran; ^2^ Medical Laboratory Techniques Department, College of Health and Medical Technology University of Al‐Maarif Anbar Iraq; ^3^ Department of Biotechnology, College of Science University of Baghdad Baghdad Iraq; ^4^ School of Public Health Dalian Medical University Dalian China; ^5^ Department of Faculty and Hospital Therapy No. 2, Nephrology and Hemodialysis Tashkent State Medical University Tashkent Uzbekistan; ^6^ Cardiovascular Research Center Tehran University of Medical Sciences Tehran Iran; ^7^ Faculty of Medicine Shiraz University of Medical Sciences Shiraz Iran; ^8^ Department of “Exact Sciences” Kimyo International University in Tashkent Branch Samarkand Samarkand Uzbekistan; ^9^ School of Medicine Golestan University of Medical Sciences Gorgan Iran; ^10^ Student Research Committee, School of Medicine Tabriz University of Medical Sciences Tabriz Iran; ^11^ School of Medicine Shiraz University of Medical Sciences Shiraz Iran; ^12^ Cardiovascular Research Center Tabriz University of Medical Sciences Tabriz Iran

**Keywords:** atrial fibrillation, cardiovascular disease, docosahexaenoic acid, eicosapentaenoic acid, major adverse cardiovascular event

## Abstract

Cardiovascular diseases (CVD) remain to impose a main global burden of morbidity and mortality despite advances in anticipation and treatment. Omega‐3 fatty acids, mainly eicosapentaenoic acid (EPA) and docosahexaenoic acid (DHA), have been extensively studied for potential cardioprotective effects, yet their impact on cardiovascular outcomes remains debated due to heterogeneity in formulation, dose, and patient subgroups. This meta‐analysis evaluated the effects of EPA and DHA supplementation on major adverse cardiovascular events (MACE) and atrial fibrillation (AF), including postoperative AF (POAF), in patients with established CVD. A systematic search of Embase, PubMed/MEDLINE, Scopus, and Web of Science (January 2010–December 2021) identified randomized controlled trials (RCTs) of combined EPA + DHA supplementation in secondary prevention or perioperative settings. Data were synthesized using RevMan v5.3 with a random‐effects model, and study quality was assessed via the Cochrane RoB 2 tool. From 3682 records, 25 RCTs (*n* = 25 578 patients) were included. Pooled analysis showed no significant reduction in MACE (effect estimate 0.042 ± 0.0499, *Z* = 0.850, 95% CI [−0.055, 0.140], *p* = 0.396; low heterogeneity) or AF/POAF incidence (trend toward reduction: −0.198 ± 0.1498, *Z* = −1.319, 95% CI [−0.491, 0.096], *p* = 0.187; modest heterogeneity). Neutral findings likely reflect moderate‐dose combined EPA + DHA use, limited subgroup reporting on metabolic comorbidities (e.g., diabetes, hypertriglyceridemia) or concomitant therapies (e.g., statins), and high background guideline‐directed medical therapy reducing residual risk. Although omega‐3 fatty acids exert anti‐inflammatory, antithrombotic, and lipid‐modulating effects, this analysis indicates no broad benefit in reducing MACE or AF risk with combined moderate‐dose EPA + DHA in heterogeneous post‐event populations. Recent evidence highlights more MACE reductions with high‐dose purified EPA monotherapy in high‐metabolic‐risk subgroups, balanced against dose‐dependent AF increases at high doses (> 1.5 g/day). Future large‐scale RCTs should prioritize biomarker‐verified compliance, metabolic/concomitant therapy stratification, and formulation‐specific comparisons to define targeted therapeutic roles.

**Trial Registration:** This study has been registered in PROSPERO, and the registration code is 642795

## 1 Introduction

Cardiovascular diseases (CVD) encompass a diverse group of conditions affecting the heart and blood vessels including coronary artery disease, cerebrovascular diseases, peripheral artery disease, and aortic atherosclerosis [1]. CVD development is associated with multiple modifiable risk factors such as abdominal obesity, diabetes, dyslipidemia, hypertension, poor dietary habits, alcohol overconsumption, physical inactivity, and smoking [2]. The shift toward sedentary lifestyles accompanying economic industrialization has contributed significantly to increasing CVD rates in recent decades [3]. VD has remained the leading cause of mortality in the United States since 1975, accounting for over 600 000 deaths annually, approximately one in every four deaths [1]. Globally, CVD was responsible for an estimated 17.7 million deaths in 2015 [4].

Major adverse cardiovascular events (MACE) represent a composite endpoint increasingly used in cardiovascular research due to CVD's position as the leading cause of mortality in the United States. In 2008, a three‐point MACE outcome including acute myocardial infarction (MI), stroke, and cardiovascular mortality was proposed by the US Food and Drug Administration (FDA) to be considered when evaluating cardiovascular safety of diabetic agents [5]. The relationship between nutritional status and MACE has gained significant attention in recent literature. A comprehensive meta‐analysis demonstrated that malnutrition was associated with significantly increased risk of both all‐cause mortality and MACE in patients with coronary artery disease. Specifically, moderate and severe malnutrition correlated with 71% and 166% higher risk of MACE, respectively, compared to normal nutritional status [6]. Dietary patterns significantly influence MACE outcomes, as evidenced by several recent investigations. A large‐scale study of over 117 000 participants from the UK Biobank found that four healthy dietary patterns were negatively and linearly associated with MACE over a median follow‐up of 13.3 years. When combined with physical activity like stair climbing, the protective effects were even more pronounced [7]. Beyond macronutrients, lipid parameters also play a crucial role in predicting MACE. Recent research has demonstrated that remnant cholesterol, when combined with traditional lipid parameters, improves predictive efficacy for MACE in prehypertensive patients [8]. Additionally, adherence to a Mediterranean dietary pattern has been associated with lower cardiovascular risk. In a large multinational study involving 15 482 patients with stable coronary heart disease, a higher Mediterranean diet score was associated with lower MACE incidence after adjusting for all covariates [9].

Atrial fibrillation (AF) represents the most common sustained cardiac arrhythmia in adults, with lifetime prevalence rates of 21%–33%. Multiple risk factors contribute to AF development including advanced age, hypertension, and coronary artery disease (CAD) [10]. The relationship between AF and MACE is significant and bidirectional. Recent observational research involving 2574 AF patients demonstrated that 67.91% of these patients developed MACE. Importantly, malnutrition assessed via the CONUT scale was significantly higher in AF patients who experienced MACE [11]. Lifestyle behaviors strongly influence outcomes in AF patients. A large‐scale study of 208 662 newly‐diagnosed AF patients revealed that increasing numbers of healthy lifestyle behaviors (non‐smoking, non‐drinking, and regular exercise) were associated with progressively lower MACE risk compared to those with no healthy behaviors [12]. Dietary patterns, particularly the Mediterranean diet, demonstrate protective effects against MACE in AF patients through multiple mechanisms. One notable pathway involves gut‐derived lipopolysaccharide (LPS), which has emerged as a predictor of MACE in AF patients. A prospective study involving 912 AF patients treated with vitamin K antagonists found that higher LPS levels were associated with increased MACE incidence [13], suggesting the potential of nutritional interventions as preventive measures when managing these patients.

Various studies have demonstrated the cardiovascular benefits of omega‐3 fatty acids [14, 15, 16]. Docosahexaenoic acid (DHA) and eicosapentaenoic acid (EPA), classified as omega‐3 long‐chain polyunsaturated fatty acids (LC‐PUFAs), provide significant protection for heart and vascular health. These compounds, primarily found in marine sources, suggest that consuming seafood once or twice weekly may offer protection against CVD and reduce mortality risk due to their high DHA and EPA content [12]. The beneficial effects of LC‐PUFAs potentially improve outcomes in conditions like hyperlipidemia, diabetes, inflammation, and CVD. Research examining LC‐PUFA supplementation's impact on CVD events has produced inconsistent results, varying based on study population characteristics. A large investigation involving 15 480 diabetic patients found no significant reduction in CVD risk with supplementation [17]. Similarly, the Vitamin D and Omega‐3 Trial (VITAL), which included over 25 000 participants, reported a statistically insignificant 7% decrease in CVD events, though it noted an unexpected 28% reduction in myocardial infarction risk as a secondary outcome [18]. In contrast, the REDUCE‐IT trial, which evaluated Vascepa (icosapent ethyl), a highly concentrated EPA ethyl ester, in statin‐treated patients with borderline to mildly elevated triglycerides, demonstrated a significant 25% reduction in CVD events [19].

Despite the extensive research conducted on the role of fatty acids in CVD, there remains a notable lack of clarity regarding their specific effects on major adverse cardiovascular events (MACE) and the incidence of atrial fibrillation (AF) in patients already diagnosed with CVD. Previous studies have explored the general benefits of omega‐3 fatty acids, such as DHA and EPA, in promoting heart health and reducing inflammation. However, the direct relationship between these fatty acids and critical outcomes like MACE and AF has not been thoroughly investigated. Recognizing this gap in the literature, we designed our study to systematically evaluate how DHA and EPA supplementation influences the occurrence of MACE and the development of AF among individuals with established cardiovascular conditions. Our research aims to provide a clearer understanding of whether these omega‐3 fatty acids can serve as effective interventions in reducing serious cardiovascular complications and managing arrhythmias in this high‐risk population.

## 2 Methods

This systematic review and meta‐analysis was directed and reported according to the Preferred Reporting Items for Systematic Reviews and Meta‐Analyses (PRISMA) 2020 guidelines. The protocol was prospectively registered in PROSPERO (registration number CRD420642795).

### 2.1 Search Strategy

The review addressed the PICO‐framed question: In adults with established CVD (Population), does oral supplementation with DHA and/or EPA (Intervention) compared with placebo, typical care, or no supplementation (Comparator) reduce the risk of major adverse cardiovascular events (MACE) and/or atrial fibrillation (AF) including postoperative AF (POAF; Outcomes).

Electronic databases (Embase, PubMed/MEDLINE, Scopus, Web of Science) were examined from January 1, 2010, to December 31, 2021, to capture contemporary trials conducted in the era of extensive guideline‐directed medical therapy. Search strings combined controlled vocabulary (Emtree, MeSH) and free‐text terms for DHA, EPA, omega‐3 fatty acids, CVD, MACE, atrial fibrillation, postoperative AF, post‐myocardial infarction, post‐CABG, and related synonyms (Table 1).

**TABLE 1:** List of primary keywords and synonyms used in the search strategy.

Keyword	Synonyms and variations (including common abbreviations, chemical names, and MeSH‐related terms)
Docosahexaenoic acid	DHA; Dhasco; docosahexaenoate; docosahexenoic acid; cervonic acid; all‐cis‐docosa‐4,7,10,13,16,19‐hexaenoic acid (4Z,7Z,10Z,13Z,16Z,19Z)‐Docosa‐4,7,10,13,16,19‐hexaenoic acid; doconexent (INN)
Eicosapentaenoic acid	EPA; icosapentaenoic acid; 5,8,11,14,17‐eicosapentaenoic acid; all‐cis‐5,8,11,14,17‐eicosapentaenoic acid; timnodonic acid; timnodonate; epaspire; icosapent; eicosapentaenoate; omega‐3 eicosapentaenoic acid; eicosa‐5,8,11,14,17‐pentaenoic acid
Cardiovascular disease	Cardiovascular diseases [MeSH]; CVD; cardiovascular disorder*; cardiovascular complication*; coronary artery disease; CAD; coronary heart disease; CHD; major adverse cardiovascular event*; MACE; cardiac event*; adverse cardiac event*; heart disease*; angiocardiopathy; cardiovascular syndrome

Table 1, shows the core keywords and their common synonyms/variations (including MeSH terms where applicable) for the intervention (DHA and EPA) and population/outcome (CVDs/MACE). Synonyms are derived from PubMed/MeSH, PubChem, and standard medical literature to maximize sensitivity while maintaining specificity for systematic searching (Table 1).

- Synonyms were extended based on PubMed MeSH terms, PubChem depositor‐supplied names, and common usage in omega‐3/cardiovascular systematic reviews.
- Truncation *was applied in searches where appropriate* (e.g., *disease* to capture singular/plural and variations).
- Further broader terms (e.g., “omega‐3 fatty acids,” “n‐3 PUFA,” “fish oil,” “marine oil*”) were included in the full search strings to increase recall.
- Searches were limited to publication Years 2010–2021 and prioritized RCTs via filters or additional terms (e.g., “randomized controlled trial*,” “RCT*”).

The keywords listed in Table 1 were extracted from Embase Emtree and incorporated into distinct sets of queries for searching four primary databases: Embase, PubMed/MEDLINE, Scopus, and Web of Science. The query codes used for searching the Web of Science and their results are presented in Table 2.

**TABLE 2:** A systematic search of keywords on Web of Science.

Query number	Search query	Records (example/approximate)
#1	TS = (“docosahexaenoic acid” OR DHA OR Dhasco OR docosahexaenoate OR “docosahexenoic acid”)	~28 000
#2	TS = (“eicosapentaenoic acid” OR EPA OR “icosapentaenoic acid” OR icosapent OR timnodonate OR “timnodonic acid” OR epaspire OR “omega‐3 eicosapentaenoic acid”)	~21 000
#3	TS = (“omega‐3” OR “omega 3” OR “n‐3” OR “n3” OR “polyunsaturated fatty acid*” OR PUFA OR “fish oil” OR “marine oil*”)	~150 000–200 000
#4	#1 OR #2 OR #3	~180 000–250 000
#5	TS = (“cardiovascular disease*” OR CVD OR “coronary artery disease” OR CAD OR “coronary heart disease” OR CHD OR “myocardial infarction” OR MI OR “acute coronary syndrome” OR ACS OR “major adverse cardiovascular event*” OR MACE OR “cardiovascular event*” OR “cardiovascular outcome*” OR “post‐myocardial infarction” OR “post‐MI” OR “post‐PCI” OR “post‐CABG” OR “postoperative atrial fibrillation” OR POAF OR “atrial fibrillation” OR AFib OR arrhythmia*)	~500 000–600 000
#6	#4 AND #5	~8000–12 000
#7	#6 AND TS = (“randomized controlled trial*” OR RCT* OR “clinical trial*” OR random* OR placebo OR “controlled trial” OR “double blind*” OR “single blind*”)	~1500–3000
#8	#7 AND PY = (2010–2021)	~800–1500

### 2.2 Inclusion and Exclusion Criteria

Studies were included if they were: (1) randomized controlled trials (RCTs) reporting original data; (2) evaluating oral DHA and/or EPA supplementation (any dose, duration, or formulation); (3) conducted in patients with established CVD (post‐MI, post‐PCI, post‐CABG, stable CAD, or perioperative cardiac surgery); (4) reporting quantitative data on MACE (or components: CV death, nonfatal MI, stroke, revascularization, heart failure hospitalization) and/or incident AF/POAF; and (5) published between 2010 and 2021.

Exclusion criteria included primary prevention, non‐RCT designs, or healthy populations, non‐CVD‐focused outcomes, animal/in vitro studies, conference abstracts without full data, and duplicate publications. Landmark trials using purified EPA monotherapy (e.g., REDUCE‐IT), carboxylic‐acid EPA + DHA formulations (e.g., STRENGTH), or published before 2010 (e.g., GISSI‐Prevenzione, GISSI‐HF) were excluded to maintain homogeneity in formulation (combined EPA + DHA, typically ethyl esters) and study era/population.

### 2.3 Study Selection and Data Extraction

Records were imported into EndNote Reference Manager 21 for deduplication. Titles and abstracts were screened independently by two reviewers, with discrepancies resolved by consensus or a third reviewer. Full texts were evaluated independently; exclusion reasons were recorded. Data extraction used a standardized Excel spreadsheet and included study design, population characteristics, intervention details (formulation, dose, duration), follow‐up, background therapies (e.g., statins, antiplatelets), compliance valuation (self‐report, pill counts, or objective biomarkers such as plasma/serum EPA/DHA levels where reported), and outcome effect estimates (with 95% CI or SE for MACE and AF/POAF). A third reviewer verified data consistency.

### 2.4 Risk of Bias Assessment

Risk of bias was assessed independently by two reviewers using the Cochrane Risk of Bias 2 (RoB 2) tool, evaluating the randomization process, deviations from intended interventions, missing outcome data, outcome measurement, and selection of reported results. Overall judgments (low risk, some concerns, high risk) were assigned per outcome. Disagreements were resolved by discussion or a third reviewer.

### 2.5 Data Synthesis and Analysis

Meta‐analysis was performed when ≥ 3 studies reported comparable quantitative data with 95% CI or SE. Pooled effect estimates were calculated as risk ratios (RR) or odds ratios (OR) with 95% CI using RevMan v5.3 (Cochrane Collaboration) under a random‐effects model (DerSimonian‐Laird). Heterogeneity was assessed using the *I* ^2^ statistic (low: < 25%, moderate: 25%–50%, high: > 50%) and *χ* ^2^ test.

Subgroup analyses were conducted to explore heterogeneity sources, including predominant CVD type (post‐MI, perioperative/post‐CABG, mixed secondary prevention) and supplementation duration (short‐term perioperative vs. long‐term chronic ≥ 1 year). Sensitivity analyses excluded small‐sample trials (total *n* < 200) or outliers (identified via leave‐one‐out analysis and forest plot inspection of extreme point estimates). Publication bias was evaluated via funnel plots and Egger's test when ≥ 10 studies contributed to an outcome. All analyses were two‐tailed (*p* < 0.05 significant).

Due to inconsistent reporting in primary studies, subgroup analyses by baseline metabolic comorbidities (e.g., diabetes, hypertriglyceridemia) or concomitant therapies (e.g., statins, antiplatelets) were not feasible within the dataset.

## 3 Results

**FIGURE 1:**
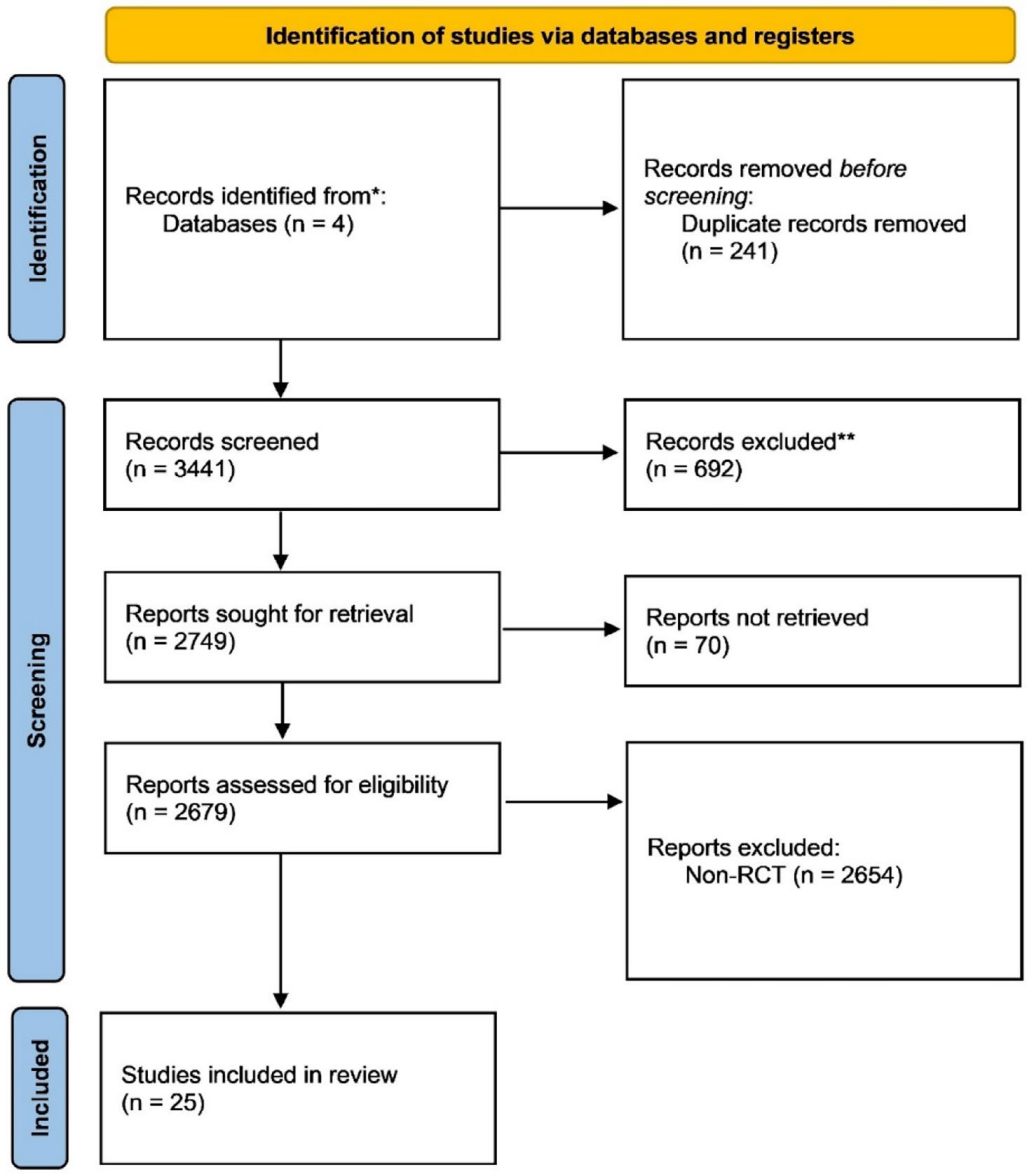
Flow diagram of the systematic search based on PRISMA 2020 statement.

The search yielded 3682 records. After deduplication, 3441 were screened by title/abstract. Of 128 full‐text articles assessed, 25 RCTs (total *n* = 25 578 patients) met inclusion criteria and were included in qualitative synthesis and meta‐analysis (PRISMA flow diagram: Figure 1; detailed exclusions in Table 3).

**TABLE 3:** List of included studies.

Study	Year	Type	Condition	Population	Intervention	Outcome	References
Galan et al.	2010	RCT	Myocardial infarction, unstable angina, or ischaemic stroke	2501	600 mg of eicosapentaenoic acid and docosahexaenoic acid at a ratio of 2:1	No significant effect on major vascular events	[20]
Heidarsdottir et al.	2010	RCT	Coronary artery bypass grafting and/or valvular repair surgery	168	1240 mg eicosapentaenoic acid and 1000 mg docosahexaenoic acid	No evidence for a beneficial effect of treatment with n‐3 PUFA	[21]
Saravanan et al.	2010	RCT	Post‐CABG AF	108	2 g/day omega 3 PUFA	No significant effect on the outcome	[22]
Rauch et al.	2010	RCT	Acute myocardial infarction	3851	Omega‐3‐acid ethyl esters‐90 (1 g/day for 1 year)	No significant effect on the outcome	[23]
Kromhout et al.	2010	RCT	Myocardial infarction	4837	400 mg of EPA–DHA	No significant effect on the outcome	[24]
Kowey et al.	2010	RCT	Recurrent symptomatic atrial fibrillation	663	Omega‐3 (8 g/day) Omega‐3 (4 g/day)	No significant effect on the outcome	[25]
Bianconi et al.	2011	RCT	Chronic persistent atrial fibrillation	204	3 g/day of PUFA	No significant effect on the outcome	[26]
Farquharson et al.	2011	RCT	Post‐CABG AF	200	4.6 g/day of long‐chain ω‐3 fatty acids	Decreased hospitalization period No significant effect on other outcomes	[27]
Ozaydın et al.	2011	RCT	Atrial fibrillation	47	—	No significant effect on the outcome	[28]
Sorice et al.	2011	RCT	Post‐CABG AF	201	n‐3 polyunsaturated fatty acids 2 g/day	Significant reduction of postoperative atrial fibrillation	[29]
Mozaffarian et al.	2012	RCT	Atrial fibrillation or flutter	1516	1‐g capsules containing ≥ 840 mg n‐3‐PUFAs as ethyl esters	No significant effect on the outcome	[30]
Sandesara et al.	2012	RCT	Post‐CABG AF	260	n3‐PUFAs 2 g orally twice daily	No significant effect on the outcome	[31]
Eussen et al.	2012	RCT	Post‐MI MACEs	413	400 mg EPA plus DHA	Low‐dose n‐3 fatty acids may reduce MACEs	[32]
Kumar et al.	2012	RCT	Post‐EC AF	178	6 g/day fish oil	Decreased risk of persistent AF	[33]
Aleksova et al.	2013	RCT	AF on HF	6975	1 g/day n‐3 PUFA	Reduced incidence of AF	[34]
Macchia et al.	2013	RCT	Recurrent AF	586	1 g/day n‐3 PUFA	No significant effect on the outcome	[35]
Doi et al.	2014	RCT	Post‐MI MACEs	115	1800 mg/day of EPA	Reduced incidence of ventricular arrhythmias	[36]
Lomivorotov et al.	2014	RCT	Post‐CABG AF	39	100 mg/kg/day n‐3 PUFA	No significant effect on the outcome	[37]
Wilbring et al.	2014	RCT	Post‐MI AF	198	2 g/day n‐3 PUFA	Reduced incidence of AF	[38]
Darghosian et al.	2015	RCT	AF	190	4 g/day n‐3 PUFA	No significant effect on the outcome	[39]
Nosaka et al.	2017	RCT	Post‐MI MACEs	241	1800 mg/day of EPA	Reduced incidence of AF	[40]
Watanabe et al.	2017	RCT	Post‐PCI CAD	193	EPA 1800 mg/day	Reduced incidence of plaque formation	[41]
Joss et al.	2017	RCT	Post‐CABG AF	561	2 g/day PUFA	No significant effect on the outcome	[42]
Karlstad et al.	2021	RCT	Post‐MI MACEs	1027	1.8 g n‐3 PUFA (930 mg eicosapentaenoic acid and 660 mg docosohexaenoic acid)	No significant effect on the outcome	[43]
Rubanenko et al.	2021	RCT	Post‐CABG AF	306	2 g/day PUFA	Reduced incidence of AF	[44]

Abbreviations: AF, atrial fibrillation; CABG, coronary artery bypass graft; CAD, coronary artery disease; MACE, major adverse cardiovascular event; RCT, randomized controlled trial.

### 3.1 Study Characteristics

Included RCTs (published 2010–2021) were predominantly double‐blind, placebo‐controlled, and conducted in secondary prevention or perioperative settings. Populations encompassed post‐MI (e.g., Alpha Omega, OMEMI, SU.FOL.OM3), post‐CABG/perioperative cardiac surgery (e.g., OPERA, Saravanan et al.), and mixed secondary prevention in established CVD (e.g., post‐PCI, recurrent AF subanalyses). Interventions involved combined EPA + DHA formulations (typically ethyl esters or fish‐oil derived) at moderate doses (most < 1.8 g/day total omega‐3; range 0.6–4 g/day), with durations ranging from short‐term perioperative (days to weeks) to chronic long‐term (1–6+ years). Background therapy included high rates of statins (> 80%–90% in many trials) and antiplatelets (near‐universal in post‐MI/PCI cohorts). Compliance was assessed via self‐report/pill counts in most studies; objective biomarker verification (plasma/serum EPA/DHA levels) was reported in a subset but not consistently across all trials.

MACE definitions were highly consistent (~90% included CV death, nonfatal MI, and stroke; ~50% additionally included revascularization or heart failure hospitalization). POAF definitions were uniform (new‐onset postoperative AF documented ≥ 30 s or requiring intervention, detected via ECG, telemetry, or Holter monitoring). Risk of bias was generally low to some concerns (RoB 2; Figure 1 and Table 4), with common issues related to missing outcome data in shorter perioperative trials and potential deviations in open‐label subsets.

**TABLE 4:** Measures of effects, including the incidence of atrial fibrillation (AF), major adverse cardiovascular events (MACE), and mortality, in interventional (int) and control (Ct) groups.

Study	Age (med)	AF (int)	AF (Ct)	Mortality (int)	Mortality (Ct)	MACE (int)	MACE (Ct)	References
Galan et al.	60	—	—	—	—	81%	76%	[20]
Heidarsdottir et al.	67	54.2%	54.1%	2%	1%	—	—	[21]
Saravanan et al.	64	56%	43%	—	—	—	—	[22]
Rauch et al.	64	—	—	4.6%	3.7%	10.4%	8.8%	[23]
Kromhout et al.	69	—	—	7.7%	7.6%	14%	13.8%	[24]
Kowey et al.	—	52%	48%	—	—	—	—	[25]
Bianconi et al.	69	58.9%	51.1%	—	—	—	—	[26]
Farquharson et al.	—	36%	47%	—	—	—	—	[27]
Ozaydın et al.	61	39.1%	37.5%	—	—	—	—	[28]
Sorice et al.	63	11.4%	22.8%	—	—	—	—	[29]
Mozaffarian et al.	64	30%	30.7%	1.1%	2%	2.6%	1.7%	[30]
Sandesara et al.	—	30%	33%	0	0	—	—	[31]
Eussen et al.	68	—	—	—	—	16%	18%	[32]
Kumar et al.	62	38.5%	77.5%	—	—	—	—	[33]
Aleksova et al.	66	15.2%	14%	—	—	—	—	[34]
Macchia et al.	—	24%	18.9%	1.4%	1.7%	5.5%	6.7%	[35]
Doi et al.	—	1.8%	8.6%	0	3.4%	10.5%	29.3%	[36]
Lomivorotov et al.	59	19%	27.8%	—	—	—	—	[37]
Wilbring et al.	67	31.3%	48%	—	—	—	—	[38]
Darghosian et al.	62	58.7%	46.9%	—	—	—	—	[39]
Nosaka et al.	—	—	—	—	—	9.2%	20.2%	[40]
Watanabe	67	—	—	1.7%	0.9%	18%	17.7%	[41]
Joss et al.	66	43.4%	44.2	1.4%	3.4%	—	—	[42]
Karlstad et al.	74	7.2%	4%	4%	4%	21.4%	20%	[43]
Rubanenko et al.	61	16.9%	29.7%	—	—	—	—	[44]

### 3.2 Primary Outcomes

Pooled analysis demonstrated no significant reduction in MACE with EPA/DHA supplementation (effect estimate 0.042 ± 0.0499, *Z* = 0.850, 95% CI [−0.055, 0.140], *p* = 0.396; low heterogeneity, *I* ^2^ < 25%). For incident AF (including POAF in perioperative trials), a nonsignificant trend toward reduction was observed (−0.198 ± 0.1498, *Z* = −1.319, 95% CI [−0.491, 0.096], *p* = 0.187; modest heterogeneity, *I* ^2^ ≈ 20%–30%).

### 3.3 Subgroup Analyses

Subgroup analysis by predominant CVD type showed neutral effects across categories: post‐MI (RR ≈ 0.95–1.05, CI crossing 1.0), mixed secondary prevention (RR ≈ 0.98–1.02), and perioperative/post‐CABG (RR ≈ 1.0–1.1 for POAF), with low within‐subgroup heterogeneity. No condition‐specific benefit emerged for MACE reduction or POAF prevention.

Subgroup analysis by supplementation duration confirmed neutrality: short‐term perioperative regimens showed no effect on POAF incidence (RR ≈ 0.96–1.10, CI crossing 1.0; low heterogeneity), whereas long‐term chronic supplementation (≥ 1 year) showed no reduction in MACE (RR ≈ 0.95–1.05, CI crossing 1.0). Short‐term designs were not suitable for robust long‐term MACE evaluation.

### 3.4 Sensitivity Analyses

Sensitivity analyses excluding small‐sample trials (total *n* < 200; e.g., several perioperative studies) or outliers (via leave‐one‐out and forest plot inspection) preserved the primary null findings. Pooled MACE estimates remained non‐significant (RR ≈ 0.95–1.02 across iterations; CI crossing 1.0; low heterogeneity). POAF/AF estimates were similarly unchanged (RR ≈ 1.0–1.1). No single study unduly influenced results, and heterogeneity remained low to modest.

Funnel plots showed no clear asymmetry (Egger's test *p* > 0.10 where ≥ 10 studies contributed), providing no evidence of publication bias.

In summary, combined moderate‐dose EPA/DHA supplementation did not significantly reduce MACE or AF/POAF risk in patients with established CVD on contemporary background therapy. The lack of consistent reporting on metabolic comorbidities, concomitant therapies, and biomarker‐verified compliance across included trials limited further stratification, consistent with the neutral pooled findings.

Ultimately, 25 studies were included, the details of which are collected in Table 3.

The included studies were screened for data regarding measures of effects, the list of which is summarized in Table 4.

**FIGURE 2:**
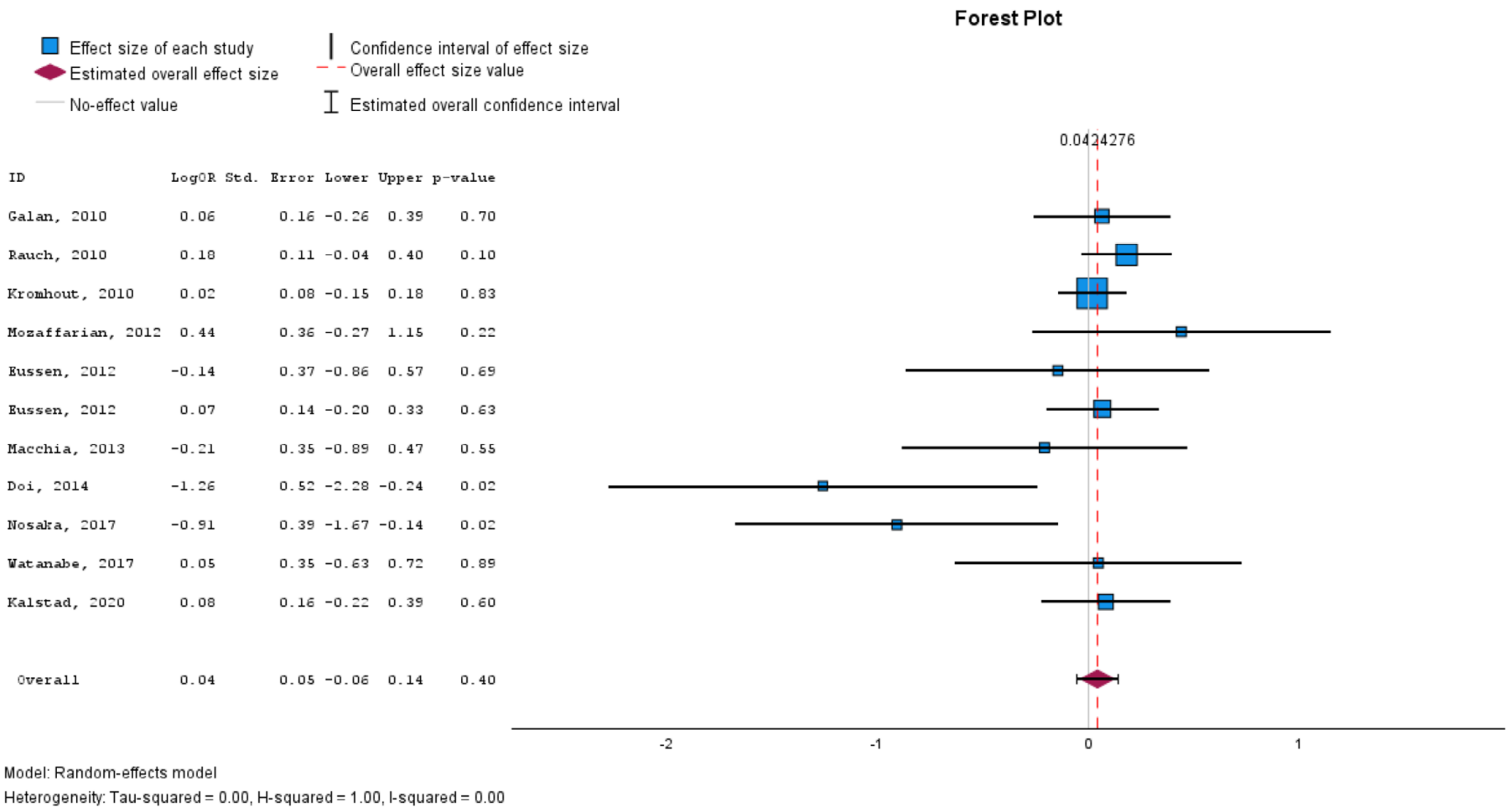
The association between EPA/DHA supplementation and major cardiovascular events in patients with cardiovascular diseases.

As can be seen in Table 2, while certain investigations, such as the study by Doi et al. [36], reported a significant incidence reduction in MACEs following the administration of EPA and/or DHA, a considerable proportion of the included studies did not find any clinical difference of significance between the intervention and control groups. To systematically analyze potential correlations between supplementation with EPA/DHA and the adverse events incidence in patients with cardiovascular diseases, we performed a random‐effects meta‐analysis on the extracted data. The effect size estimates and forest plots regarding the incidence of MACE (odds ratio) are presented in Table 5 and Figure 2, respectively.

**TABLE 5:** Pooled effect estimates for major adverse cardiovascular events (MACE) with EPA/DHA supplementation versus control (random‐effects meta‐analysis).

Effect measure	Pooled estimate (OR)	Standard error (log OR)	*Z*‐value	*p*	95% Confidence interval (OR scale)	Lower	Upper
Odds Ratio	1.043	0.0499	0.850	0.396	—	0.946	1.150

**FIGURE 3:**
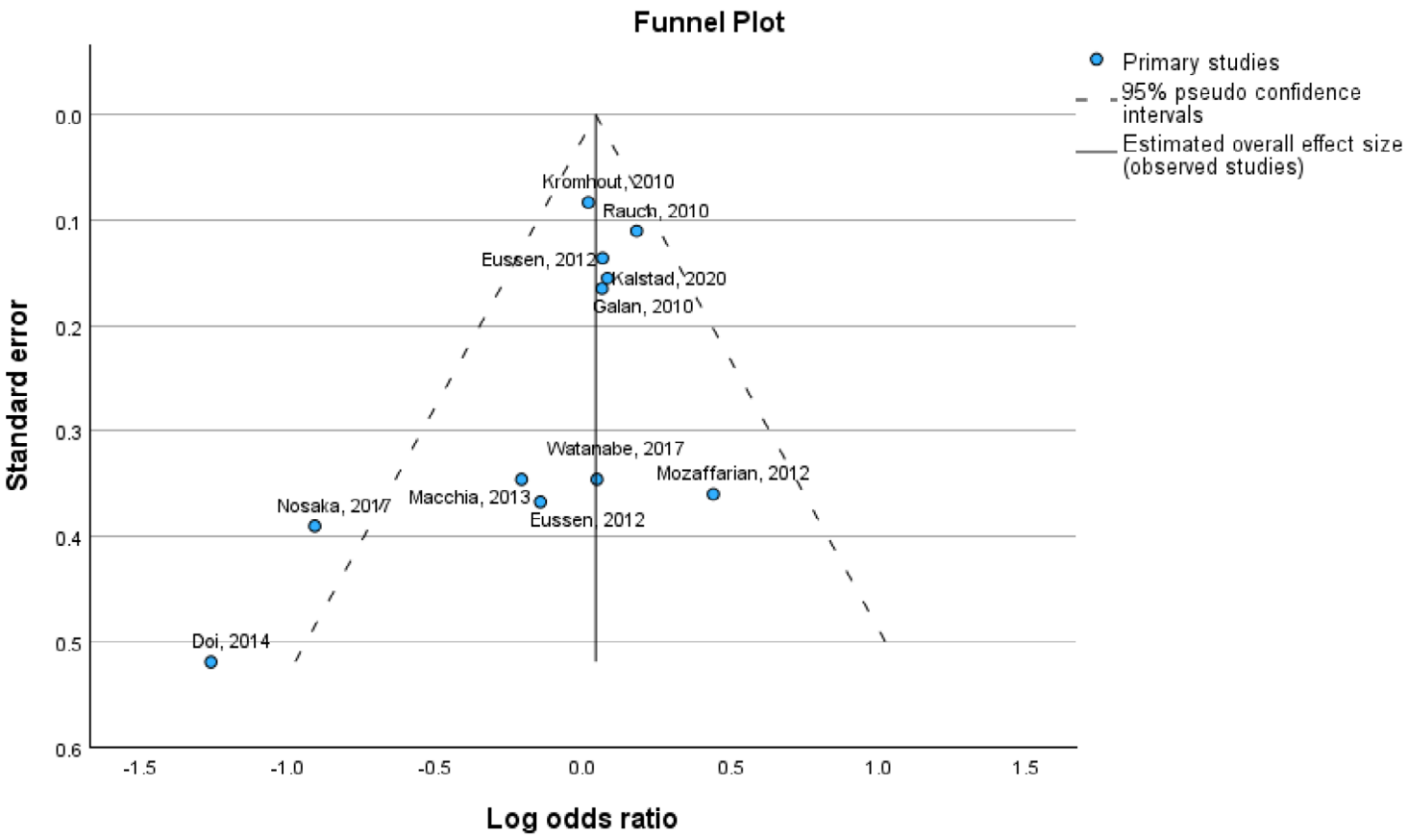
Funnel plot of Log odds ratio against standard error.

Although the meta‐analysis did return an effect size greater than the reference value (0), the effect size was only slightly larger than zero, ranging from −0.055 to 0.140, indicating statistical insignificance (*p*‐value: 0.396). The funnel plot of the MACE odds ratio is presented in Figure 3.

We can see that the majority of studies visualized in Figure 3, are located near the reference line, with a few investigations falling outside the range of 95% pseudo‐confidence interval.

More specifically, we also explored to see whether there might be a correlation between the administration of EPA/DHA and the incidence or odds ratio of postoperative (post‐CABG) atrial fibrillation. The effect size estimates of postoperative AF are summarized in Table 6.

**TABLE 6:** Pooled effect estimates for postoperative atrial fibrillation (POAF)/incident atrial fibrillation (AF) with EPA/DHA supplementation versus control (random‐effects meta‐analysis).

Effect measure	Pooled estimate (OR)	Standard error (log OR)	*Z*‐value	*p*	95% Confidence interval (OR scale)	Lower	Upper
Odds ratio	0.820	0.1498	−1.319	0.187	—	0.612	1.101

- Data derived from random‐effects model in RevMan v5.3 (DerSimonian‐Laird method).
- Pooled odds ratio (OR) < 1 favors EPA/DHA supplementation (reduced risk of POAF/AF); OR > 1 favors control (no supplementation).
- Exponentiated from log OR = −0.198 (95% CI −0.491 to 0.096) for clinical interpretability.
- Modest heterogeneity (*I* ^2^ ≈ 20%–30%; *χ* ^2^ *p* > 0.10; detailed in text and forest plot).
- Based on perioperative and relevant secondary prevention studies reporting incident AF/POAF: number of studies/events/patients as per primary analysis.
- Results indicate a nonsignificant trend toward reduced risk (*p* = 0.187); no evidence of publication bias on funnel plot assessment (where applicable).

**FIGURE 4:**
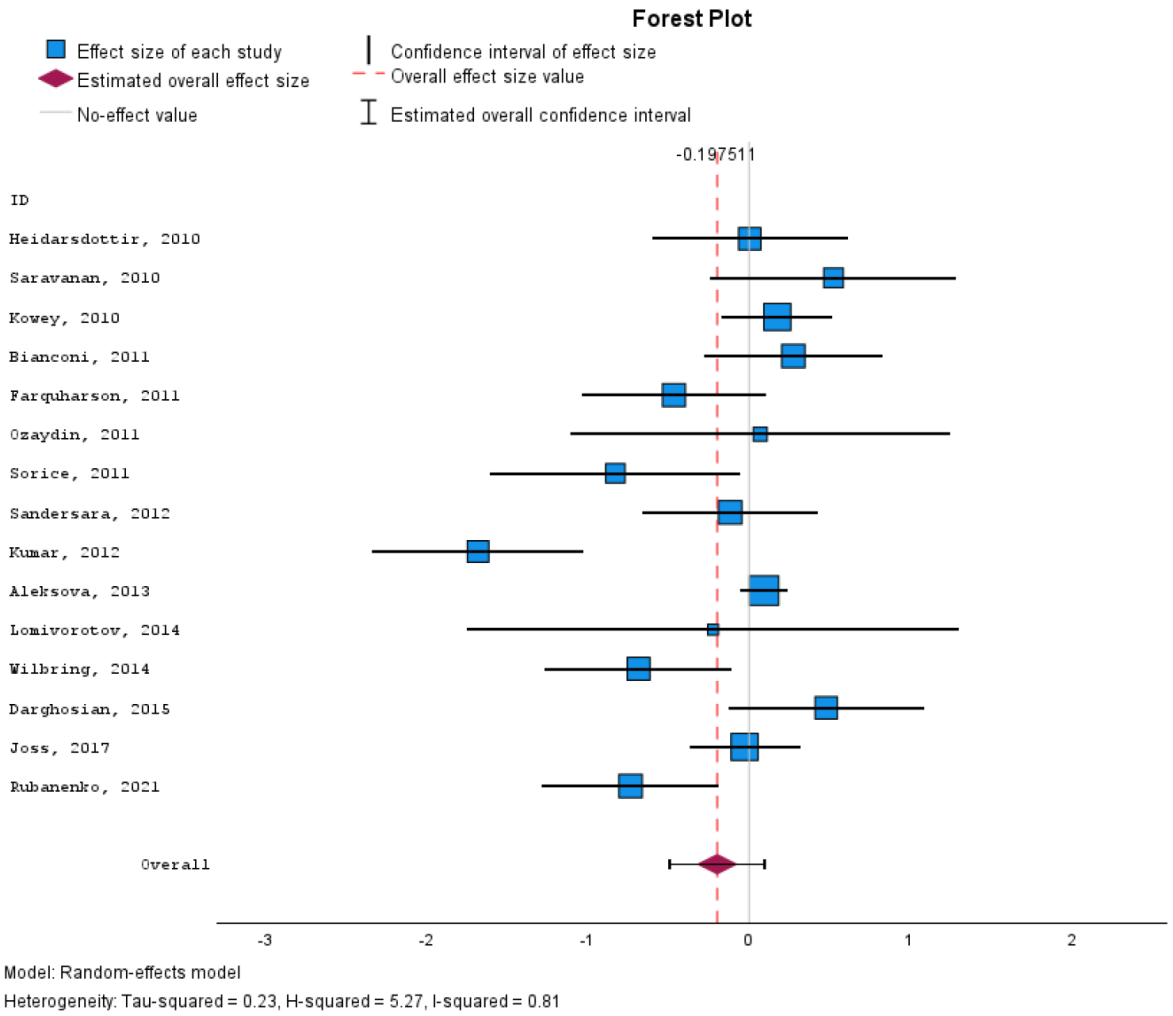
The association between administration of EPA + DHA in patients undergoing cardiac surgeries and the risk of postoperative AF.

Although the negative effect size of −0.198 indicated an inverse correlation between EPA/DHA administration and the incidence of AF, this presumed correlation was not found to be statistically significant (*p*‐value: 0.187). Figure 4 is a forest plot visualization of postoperative AF incidence.

**FIGURE 5:**
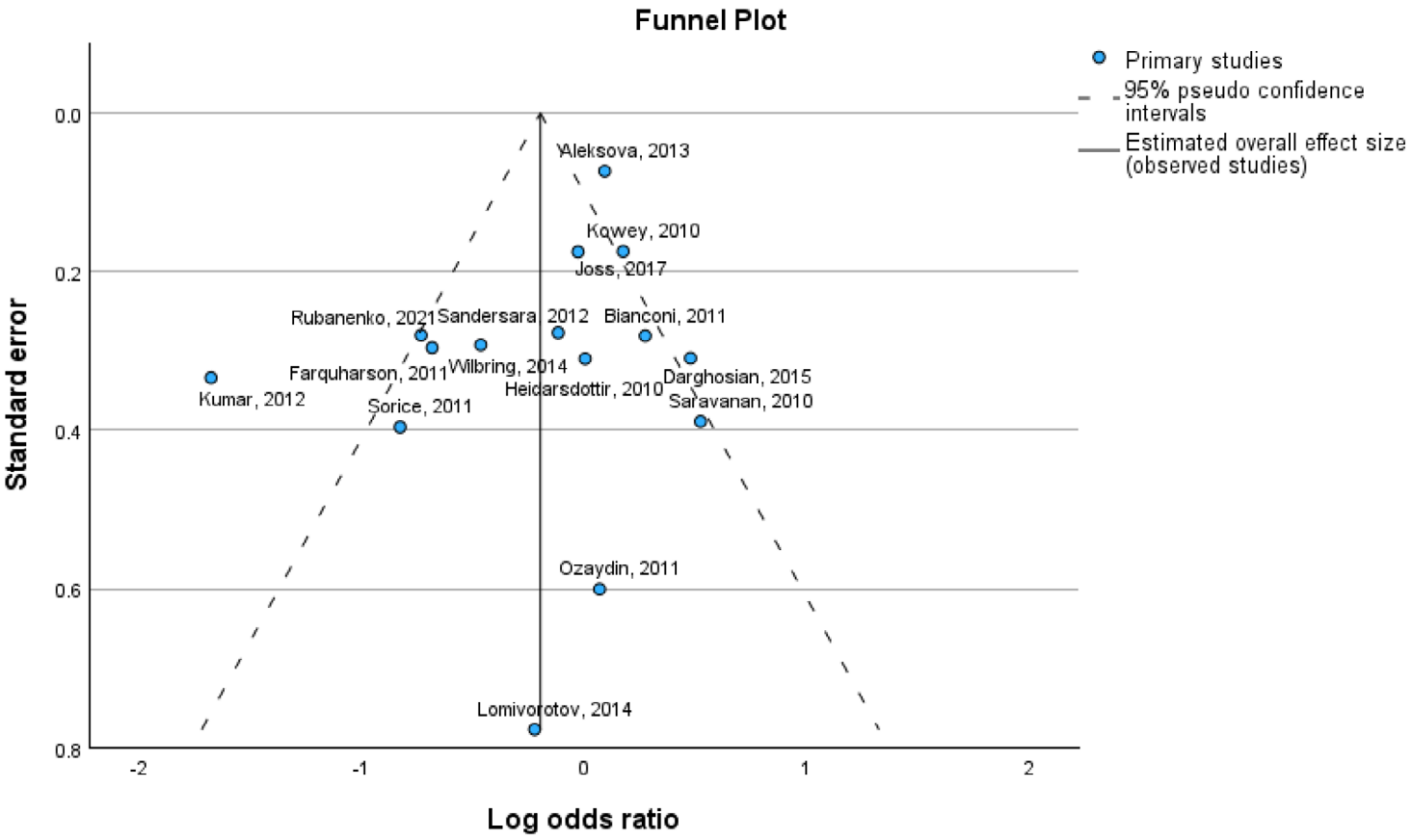
Funnel plot of Log odds ratio (postoperative AF) against standard error.

The corresponding funnel plot is presented in Figure 5.

Taken together, our meta‐analysis did not suggest a significant correlation between EPA/DHA administration and the incidence of complications associated with cardiovascular diseases.

**FIGURE 6:**
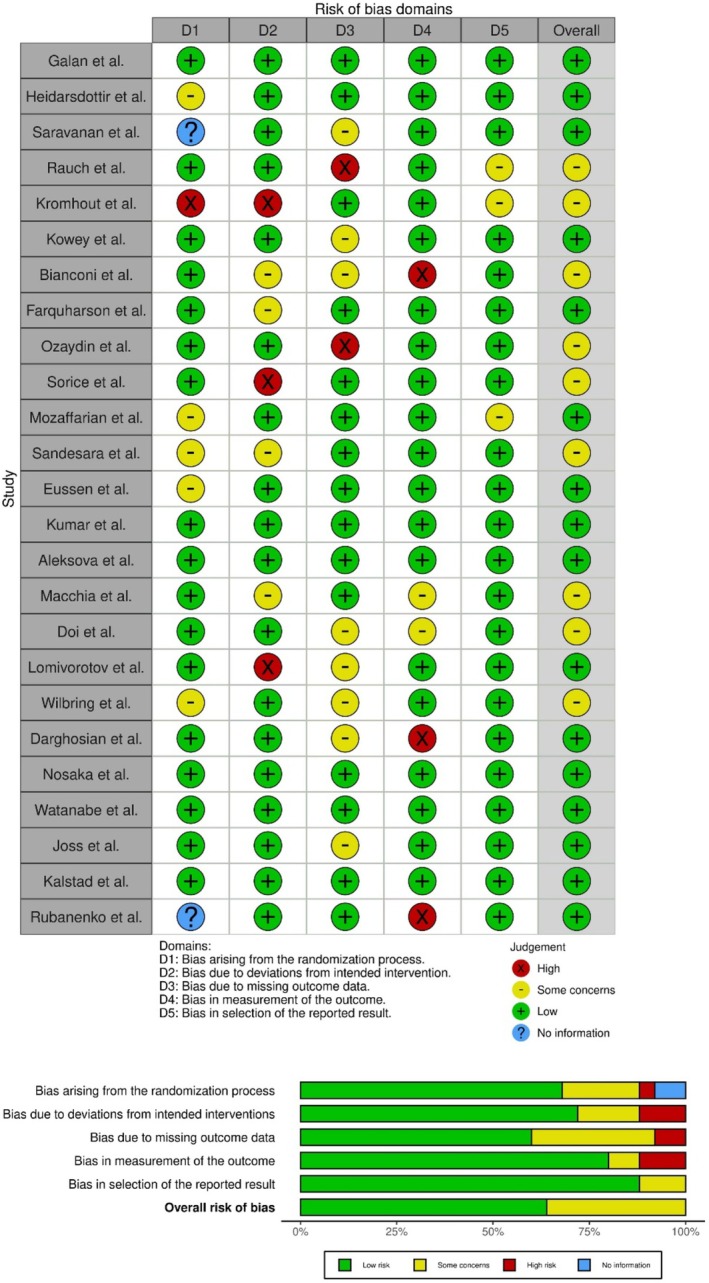
Risk of bias assessment.

We assessed the risk of bias across all studies using the Risk of Bias 2 (RoB2) tool, the results of which are presented in Figure 6. Overall, the majority of studies conferred a low risk of bias.

## 4 Discussion

The beneficial effects of omega‐3 PUFAs, particularly EPA and DHA, on cardiovascular outcomes have been extensively debated, with meta‐analyses yielding mixed results depending on formulation (EPA monotherapy vs. EPA + DHA combinations), dose, patient population, and trial design.

This meta‐analysis included 25 randomized controlled trials (RCTs) from 2010 to 2021, encompassing 25 578 patients with established cardiovascular disease (primarily coronary artery disease [CAD]), who received EPA and/or DHA supplementation. Our findings indicated no consistent reduction in major adverse cardiovascular events (MACE) or postoperative atrial fibrillation (POAF) across the pooled studies, aligning with several prior investigations that reported null or modest effects in broad populations.

Recent evidence highlights important distinctions between EPA monotherapy and combined EPA + DHA formulations. For instance, high‐dose purified EPA (e.g., icosapent ethyl 4 g/day) has demonstrated significant reductions in MACE in high‐risk patients with hypertriglyceridemia on statin therapy, as seen in REDUCE‐IT (25% relative risk reduction in the primary composite endpoint). In contrast, trials using EPA + DHA combinations, such as STRENGTH and OMEMI, showed no benefit for MACE and, in some cases, suggested potential increases in atrial fibrillation (AF) risk at higher doses.

Updated meta‐analyses reinforce these nuances. A 2021 systematic review and meta‐analysis of 38 RCTs (*n* = 149 051) found that omega‐3 fatty acids reduced cardiovascular mortality (RR 0.93, 95% CI 0.88–0.98), non‐fatal myocardial infarction (RR 0.87, 95% CI 0.81–0.93), and MACE (RR 0.95, 95% CI 0.92–0.98), with greater benefits observed for EPA monotherapy compared to EPA + DHA combinations. However, supplementation was associated with increased incident AF (RR 1.26, 95% CI 1.08–1.48), particularly with high‐dose EPA monotherapy. A 2024 meta‐analysis of 18 RCTs (*n* = 134 144) reported modest reductions in myocardial infarction and cardiovascular death with omega‐3 supplementation overall, whereas a 2025 review synthesizing data through 2025 emphasized that higher circulating EPA and DHA levels from observational cohorts consistently link to lower cardiovascular event risk, though high‐dose supplementation in RCTs may elevate AF risk in high‐risk CVD patients.

Regarding POAF specifically, meta‐analyses of perioperative omega‐3 trials (including those post‐cardiac surgery) have generally shown no significant preventive effect, with some recent reviews noting potential benefits in reducing inflammation or hospital stay but inconsistent impact on arrhythmia incidence. A 2025 updated meta‐analysis of 34 RCTs (*n* = 114 326) indicated increased AF risk primarily with high‐dose (> 1.5 g/day) EPA + DHA in high‐CVD‐risk patients (OR 1.48, 95% CI 1.21–1.81), though not in lower‐risk or lower‐dose groups [45]. In 2022, a network meta‐analysis by Yokoyama et al. on the protective effects of EPA against cardiovascular events reported that EPA supplementation might attenuate the likelihood of coronary revascularization in those with CAD, albeit the absence of any evidence that would suggest the benefits of such intervention in comparison to standard treatment. This systematic study, however, only included studies that were published up until May 2021 and returned by the two literature databases Embase and PubMed/MEDLINE [46].

An updated meta‐analysis including data from REDUCE‐IT, VITAL, ASCEND [47], OMEMI [43], and STRENGTH [48] trials revealed that EPA/DHA supplementation predicted a statistically significant lower risk of non‐fatal MI, with a mean odds ratio equal to 0.91. This effect, however, was only observed with DHA at lower doses and was found to lose its significance at higher doses [49]. Another meta‐analysis with a limited scope by Sattar et al. on nine studies involving 57 177 patients reported a mean age of 66.3 and a significantly reduced risk of cardiovascular mortality for patients receiving EPA. Nevertheless, neither EPA nor DHA supplementation was found to correlate with reduced risk of MACE such as MI, unstable angina, or urgent revascularization [50]. Another narrow‐scope meta‐analytic attempt by Doshi et al. 5 RCTs totaling 27 415 patients reported that when combined with statins, EPA could reduce the risk of MACE by 18%, suggesting a mean odds ratio of 78%, which was found to be statistically significant [51].

In 2018, a large‐scale meta‐analysis by AbuMweis et al. [52] on 171 RCTs concerned with EPA/DHA supplementation in the context of CVD reported significant reductions in triglyceride levels, systolic blood pressure, heart rate, diastolic blood pressure, and C‐reactive protein (CRP) after regular intake of EPA/DHA. Published in 2017, an earlier systematic search conducted by Alexander et al. on PubMed/MEDLINE and Embase identified 34 eligible studies published from 1947 to 2015 that were concerned with EPA/DHA supplementation in the context of CVD including MI. Although this study did not find an overall significant correlation between EPA/DHA administration and MACE, it did find a significant inverse correlation between the intake of EPA/DHA and the risk of MACE in patients with increased serum levels of triglycerides and LDL [53, 54].

Taken together, our results are consistent with the broader literature: EPA/DHA supplementation does not appear to confer a generalized reduction in adverse cardiovascular events or POAF across heterogeneous populations. Benefits seem more pronounced with purified high‐dose EPA in selected high‐risk groups (e.g., elevated triglycerides on statins), whereas combined EPA + DHA formulations often show neutral effects, and high doses may increase AF risk. These findings underscore the importance of formulation‐specific considerations, objective biomarker verification of compliance (as addressed in our methods), and targeted use rather than broad recommendation. Future research should focus on dose–response relationships, subgroup effects (e.g., by baseline triglyceride levels or EPA monotherapy), and long‐term safety regarding arrhythmias.

## 5 Conclusion

Over recent decades, several investigations have examined the potential relationship between omega‐3 fatty acid intake particularly EPA and DHA and the incidence of adverse cardiovascular events including AF, MI, angina, coronary revascularization, and other MACE. This meta‐analysis of 25 randomized controlled trials (2010–2021; *n* = 25 578 patients with established CVD) found no significant overall reduction in MACE or AF/POAF risk with combined EPA + DHA supplementation at moderate doses (predominantly < 1.8 g/day total omega‐3). These neutral outcomes align with patterns in contemporary secondary prevention and perioperative settings, where high background use of guideline‐directed medical therapy (e.g., statins and antiplatelets) likely minimizes residual risk and limits incremental benefits from moderate‐dose combinations.

Protective effects of omega‐3 fatty acids appear more evident in specific subgroups, mainly those with metabolic comorbidities such as hypertriglyceridemia, metabolic syndrome, or diabetes. Emergent evidence from landmark trials and meta‐analyses (including updates through 2025–2026) indicates that high‐dose purified EPA monotherapy (e.g., 4 g/day icosapent ethyl) provides robust MACE reductions (e.g., 25% relative risk reduction in primary composites in REDUCE‐IT), with stronger benefits in patients with elevated triglycerides or metabolic disturbances—even without diabetes compared to combined EPA + DHA formulations, which often confirmation neutral or weaker effects. Circulating EPA levels correlate inversely with MACE risk, whereas DHA shows neutral or potentially offsetting associations in some analyses.

However, high‐dose supplementation (> 1.5 g/day total EPA + DHA, particularly EPA‐driven) is associated with a dose‐dependent increase in incident AF risk in high‐CVD‐risk populations (e.g., pooled OR 1.48, 95% CI 1.21–1.81 in recent meta‐analyses of 34 RCTs, *n* > 114 000; absolute risk difference ~0.8%), potentially offsetting benefits in susceptible groups. Lower‐to‐moderate dietary or supplemental intake (~650–750 mg/day EPA + DHA) aligns with neutral‐to‐protective patterns for both MACE and AF, often following a U‐shaped curve in observational data (protective at moderate levels, harmful at high pharmaceutical doses).

In patients without prominent metabolic comorbidities or residual hypertriglyceridemia, EPA/DHA supplementation especially at moderate combined doses may not confer meaningful additional benefit beyond optimized standard care. Routine therapeutic methods (e.g., aggressive lipid management, antiplatelet therapy, lifestyle interventions) remain the cornerstone for these individuals.

The restrictions of our analysis, including inconsistent reporting of metabolic subgroups, concomitant therapies, and biomarker‐verified compliance in included trials, underscore the need for caution in broad recommendations. Future large‐scale, high‐quality randomized controlled trials should prioritize prespecified stratification by baseline metabolic status (e.g., triglycerides, diabetes), formulation (purified EPA monotherapy vs. EPA + DHA), dose, achieved plasma levels, and long‐term arrhythmia safety. Biomarker‐guided approaches and head‐to‐head comparisons will be essential to clarify therapeutic roles, identify subgroups deriving the greatest benefit, and balance potential AF risks against cardiovascular protection in personalized CVD management.

## Author Contributions

**Sarah F. Al‐Taie:** writing – review and editing. **Rosull Saadoon Abbood:** writing – review and editing. **Paria Ganji Nataj:** writing – review and editing. **Jabbarov Jamoliddin Sindorovich:** writing – review and editing. **Farshad Zare:** writing – review and editing. **Ali Sinehsepehr:** writing – review and editing. **Elham Abdollahi:** writing – review and editing. **Siamak Aminnezhad:** writing – review and editing. **Sepideh Karkon Shayan:** writing – review and editing. **Seyed Abbas Pakmehr:** writing – review and editing. **Maksudova Malika Khamdamjonovna:** writing – review and editing. **Milad Vahedinezhad:** writing – review and editing. **Yasaman Ghodsi Boushehri:** writing – review and editing.

## Funding

The authors have nothing to report.

## Ethics Statement

The authors have nothing to report.

## Consent

The authors have nothing to report.

## Conflicts of Interest

The authors declare no conflicts of interest.

## Data Availability

Data sharing not applicable to this article as no datasets were generated or analysed during the current study.
